# Supplementary material for: Observing collisions beyond the secular approximation limit
Source: Nat Commun. 2019 Dec 18;10:5780. doi: 10.1038/s41467-019-13706-0 (PMC6920365; doi:10.1038/s41467-019-13706-0)
Supplement: Supplementary file 1 — Supplementary Information [file 41467_2019_13706_MOESM1_ESM.pdf]

Supplementary Information for

"Observing collisions beyond the secular approximation limit"

Ma et al.

Supplementary Note 1: On the Markovian approximation

The Markovian approximation for rotational relaxation and decoherence processes assumes that the quantity of interest (here the time dependence, through the time evolution of the density matrix, of the alignment factor) can be modeled by considering that all collisions are complete. Also called impact approximation, it thus neglects the memory effects associated with those collisions that are on-going at the time (t=0) of the excitation of the system or at that ($t=2\tau_{12}$, for the echo) where the system is observed. This implicitly assumes that the relative number of on-going collisions is very small and/or that they have an effect over a very short interval of time. For the system of N_2O gas highly diluted in He investigated in the present study, both criteria are well satisfied so that the Markovian approximation is valid. Indeed, the N_2O-He accurate ab initio intermolecular potential [1] has an almost negligible well (with respect to the mean kinetic energy at room temperature). The repulsive front is relevant (i.e. has values between 0 and 600 K) in the [R_0-ΔR, R_0] range of intermolecular distances where R_0 varies between 2.7 and 4.2 Å depending on the respective orientations of the molecular and intermolecular axes and ΔR is typically 0.5 Å. This means that, for a N_2O molecule to be colliding with a He atom, the latter should be in the interval between the two spheres centered on the N_2O center of mass and of typical radii 3.0 and 3.5 Å. This corresponds to a very small volume (about 70 $Å^3$) that statistically contains very few He atoms unless the gas density is very high [for 15 amagat which corresponds to 4 10^{26} $atom.m^{-3}$, this statistical number is about 0.025). In addition, the mean relative speed of N_2O-He collisions at 295 K is 1300 $m.s^{-1}$, which implies that the distance between the two spheres (0.5 Å) is, on average, traveled in 0.038 ps. The above given numbers show that non-Markovian effects are of very small amplitude and only exist at time scales much shorter than all those involved in the present study. Assuming Markovian collisions is thus a very good approximation for the N_2O-He system but this would not be the case for pairs of colliders involving much smaller relative speeds and/or intermolecular forces at much longer ranges.

Supplementary Note 2: On the validity of the secular approximation

Consider a case where coherences have been instantaneously generated by laser pulse at t=0. This creates non-zero off-diagonal elements $\rho_{JM,J'M}(t=0^+)$, which, in the absence of collisions, have the expression:

$$\rho_{JM,J'M}(t)=\rho_{JM,J'M}(t=0^+)\exp\left[-\mathrm{i}(E_J-E_{J'})t/\hbar\right]. \quad (1)$$

Let us now assume that collisions induce transfers between coherences and, in particular, from $\rho_{J_1M_1,J_1'M_1}(t)$ to $\rho_{J_0M_0,J_0'M_0}(t)$ with a real-valued density-normalized rate $K_{J_1M_1,J_1'M_1\to J_0M_0,J_0'M_0}$. For a gas density d, the total change of $\rho_{J_0M_0,J_0'M_0}(t)$ due to transfers from $\rho_{J_1M_1,J_1'M_1}(t)$ at all possible times $t_0>0$ is:

$$\Delta\rho_{J_0M_0,J_0'M_0}(t)=K_{J_1M_1,J_1'M_1\to J_0M_0,J_0'M_0}d\times\int_0^t\rho_{J_1M_1,J_1'M_1}(t_0)\mathrm{e}^{-\mathrm{i}(E_{J_0}-E_{J_0'})(t-t_0)/\hbar}dt_0 \quad . \quad (2)$$

If we assume that these transfers are relatively small, we can consider that $\rho_{J_1M_1,J_1'M_1}(t_0)$ remains unchanged when t_0 varies so that Supplementary Equation (1) can be used and Supplementary Equation (2) becomes:

$$\begin{aligned}\Delta\rho_{J_0M_0,J_0'M_0}(t)=\rho_{J_1M_1,J_1'M_1}(t=0^+)K_{J_1M_1,J_1'M_1\to J_0M_0,J_0'M_0}d\\ \times\exp\left[-\mathrm{i}(E_{J_0}-E_{J_0'})t/\hbar\right]\int_0^t\mathrm{e}^{-\mathrm{i}(E_{J_1}-E_{J_1'}-E_{J_0}+E_{J_0'})t_0/\hbar}dt_0\end{aligned} \quad , \quad (3)$$

i.e., using Supplementary Equation (1) again

$$\frac{\Delta\rho_{J_0M_0,J_0'M_0}(t)}{\rho_{J_0M_0,J_0'M_0}(t)} = A\times d\,\frac{1-\exp\left[-\mathrm{i}(E_{J_1}-E_{J_1'}-E_{J_0}+E_{J_0'})t/\hbar\right]}{\mathrm{i}(E_{J_1}-E_{J_1'}-E_{J_0}+E_{J_0'})/\hbar} \quad , (4)$$

with

$$A \equiv \frac{\rho_{J_1M_1,J_1'M_1}(t=0^+)}{\rho_{J_0M_0,J_0'M_0}(t=0^+)} K_{J_1M_1,J_1'M_1\to J_0M_0,J_0'M_0} \quad . (5)$$

For a study of the relative evolution of this quantity with time, one must normalize the effect of collisions, which implies that:

$$d\times t = \text{Constant} \equiv \delta \text{ , (6)}$$

so that Supplementary Equation (4) becomes:

$$\mathrm{Re}\left[\frac{\Delta\rho_{J_0M_0,J_0'M_0}(t)}{\rho_{J_0M_0,J_0'M_0}(t)}\right] = B\mathrm{sinc}\left[(\omega_{J_1J'_1}-\omega_{J_0J'_0})t\right] \text{ , (7)}$$

where Re[…] denotes the real part, sinc[…] designates a cardinal sine, $\omega_{JJ'} \equiv (E_J - E_{J'})/\hbar$ and $B \equiv \delta.A$.

Supplementary Note 3: Rates of population and coherence transfers

3.1 Infinite Order Sudden model

The elements $\langle J_1M_1, J'_1M_1|\mathbf{\Lambda}|J_0M_0, J'_0M_0\rangle$ of the matrix describing losses and transfers among populations [$\rho_{JM,JM}(t)$] and coherences [$\rho_{JM,J'M}(t)$ with $J \neq J'$] were computed using the Infinite Order Sudden Approximation (IOSA). Recall that the IOSA is obtained [2] from the Close-Coupling model by first making the Centrifugal Sudden (or Coupled States CS) approximation, i.e. by assuming an effective orbital momentum eigenvalue and freezing the centrifugal potential. Then, starting from the CS equations, the IOSA freezes the molecular rotation during each collision, which corresponds to neglecting the energy difference between different rotational states. For molecule-atom collisions, one can then separate the collisional cross-sections into spectroscopic and dynamical factors [3,4]. For the N_2O-He collisions considered here, this is valid thanks to the high value of the N_2O-He pair relative translational speed and the fact that the intermolecular forces for this system are significant only in a very narrow range of intermolecular distances [1] (see also Supplementary Note 1). Within this frame, the relaxation matrix elements describing collisional exchanges among and between populations and coherences are given by [5,6]:

$$\langle J_1M_1, J'_1M_1|\mathbf{\Lambda}|J_0M_0, J'_0M_0\rangle = -\sqrt{(2J_1+1)(2J'_1+1)(2J_0+1)(2J'_0+1)}$$
$$\sum_L \begin{pmatrix} J_0 & J_1 & L \\ 0 & 0 & 0 \end{pmatrix}\begin{pmatrix} J'_0 & J'_1 & L \\ 0 & 0 & 0 \end{pmatrix}\begin{pmatrix} J_0 & J_1 & L \\ M_0 & -M_1 & M_1-M_0 \end{pmatrix}\begin{pmatrix} J'_0 & J'_1 & L \\ M_0 & -M_1 & M_1-M_0 \end{pmatrix}(2L+1)Q_L \text{ , (8)}$$

where (:::) is a 3J symbol and Q_L is the rate of collisional de-excitation from level J=L to level J=0. As widely done before for other types of studies [7,8], the latter are modelled using the exponential power gap law based on three parameter (A,α,β), i.e.:

$$Q_{L\neq 0} = A\left[L(L+1)\right]^{-\alpha}\exp(-\beta E_L/k_\mathrm{B}T) \text{ , (9)}$$

where k_B is the Boltzmann constant and, according to Ref. [9]:

$$Q_0 = -\sum_{L\neq 0}(2L+1)Q_L \text{ . (10)}$$

Note that this convention for the L=0 term differs from that used in our previous studies [7,10].

3.2 Input data

As widely done before [7,11], the parameters of Supplementary Equation (9) were here deduced from fits of pressure broadening coefficients of infrared lines. Indeed, for the $J_\mathrm{i} \rightarrow J_\mathrm{f}$ optical transition, this coefficient $\gamma_{J_\mathrm{i}J_\mathrm{f}}$ is, within the IOSA, given by [7,9,12]:

$$\gamma_{J_\mathrm{i}J_\mathrm{f}} = -Q_0 + (2J_\mathrm{i}+1)(2J_\mathrm{f}+1) \times \sum_{L\neq 0} \begin{pmatrix} J_\mathrm{i} & J_\mathrm{i} & L \\ 0 & 0 & 0 \end{pmatrix} \begin{pmatrix} J_\mathrm{f} & J_\mathrm{f} & L \\ 0 & 0 & 0 \end{pmatrix} \begin{Bmatrix} J_\mathrm{i} & J_\mathrm{f} & 1 \\ J_\mathrm{f} & J_\mathrm{i} & L \end{Bmatrix} \times (2L+1) Q_L \quad , (11)$$

where {:::} is a 6J symbol. The least-square fit of the measured He-broadened widths of N_2O lines of Ref. [13] using Supplementary Equations (9)-(11) leads to A=22.8 10^{-3} $cm^{-1}atm^{-1}$ (4.66 10^{-3} $ps^{-1}amagat^{-1}$), α=1.05, and β=0.04 with the good agreement shown in Supplementary Figure 1. Note that only the even values of L are retained in Supplementary Equations (8), (9), and (11), as done in Ref. [11], since the N_2O-He intermolecular potential is almost insensitive to a rotation by π of the N_2O molecule [1].

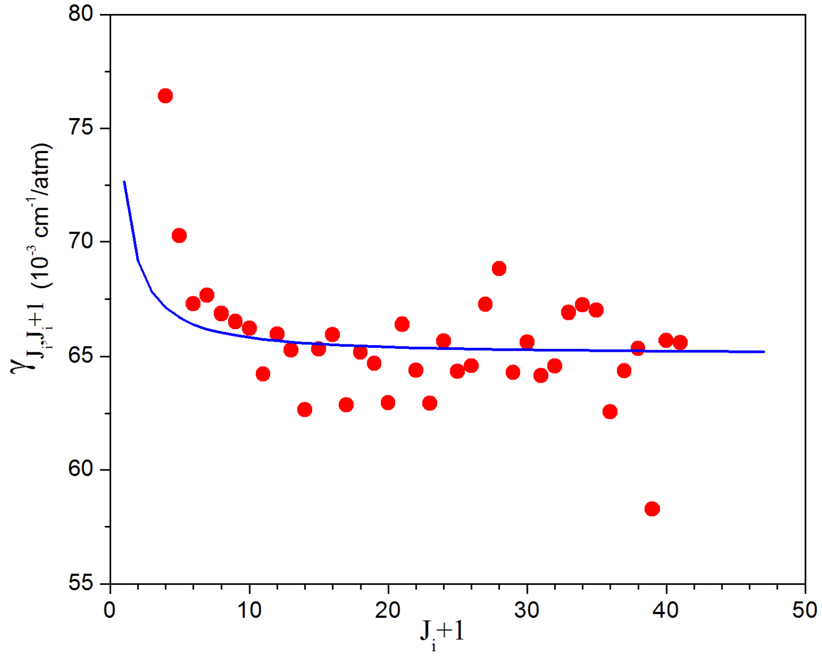

Supplementary Figure 1: Pressure broadening of N_2O lines by He. Comparison between measured line broadening coefficients of N_2O by He for infrared $\mathrm{R}(J_i \rightarrow J_i+1)$ lines [13] (symbols) [converted from 303 K to 295 K by a factor $(303/295)^{1/2}$] with those fitted with Supplementary Equations (9)-(11) (line).

3.3 From nonsecular to secular

Within the secular approximation, only the terms $\langle JM, J'M | \mathbf{\Lambda} | JM, J'M \rangle$ driving the loss of coherence ($J \neq J'$) and population ($J = J'$) and those $\langle J'M', J'M' | \mathbf{\Lambda} | JM, JM \rangle$ associated with exchanges between the $|JM\rangle$ and $|J'M'\rangle$ populations are kept. Let us first consider the exchanges between populations, driven by $\langle J'M', J'M' | \mathbf{\Lambda} | JM, JM \rangle$ with $|JM\rangle \neq |J'M'\rangle$. The associated rate is straightforwardly obtained from Supplementary Equation (8):

$$K_{J,M\to J',M'}=-\langle J'M',J'M'|\mathbf{\Lambda}|JM,JM\rangle$$
$$=(2J+1)(2J'+1)\sum_{L\neq 0}\begin{pmatrix} J & J' & L \\ M & -M' & M'-M \end{pmatrix}^2 \begin{pmatrix} J & J' & L \\ 0 & 0 & 0 \end{pmatrix}^2 (2L+1)K(L\to 0) \quad , (12)$$

which is identical to Equation (A2) of Ref. [10]. Now concerning the rate $\langle JM,JM|\mathbf{\Lambda}|JM,JM\rangle$ driving the loss of the $|JM\rangle$ population, it can be transformed using 3J properties and Supplementary Equation (10), leading to

$$\langle JM,JM|\mathbf{\Lambda}|JM,JM\rangle=\sum_{(J',M')\neq(J,M)} K_{J,M\to J',M'} \quad , (13)$$

in agreement with Equation (19) of Ref. [10]. Finally, the loss of the $|JM,J'M\rangle$ coherence is driven by the term $\langle JM,J'M|\mathbf{\Lambda}|JM,J'M\rangle$. Again using properties of the 3J symbols and Supplementary Equation (10), it can be rewritten as:

$$\langle JM,J'M|\mathbf{\Lambda}|JM,J'M\rangle=\frac{1}{2}\left[\sum_{(J'',M'')\neq(J,M)} K_{J,M\to J'',M''}+\sum_{(J'',M'')\neq(J',M)} K_{J',M\to J'',M''}\right]$$
$$+\sum_{L\neq 0}\frac{1}{2}\left\{(2J+1)\begin{pmatrix} J & L & J \\ 0 & 0 & 0 \end{pmatrix}\begin{pmatrix} J & L & J \\ -M & 0 & M \end{pmatrix}-(2J'+1)\begin{pmatrix} J' & L & J' \\ 0 & 0 & 0 \end{pmatrix}\begin{pmatrix} J' & L & J' \\ -M & 0 & M \end{pmatrix}\right\}^2 (2L+1)Q_L \quad , (14)$$

which is identical to Equations (12) and (16) of Ref. [10]. Finally note that assuming that $K_{J,M\to J',M'}$ is independent of *M* and *M'* leads [10] to the equations used in Refs. [14,15].

Supplemenraty References

1. Wang, L., Xie, D. , & Le Roy, R. J. New four-dimensional ab initio potential energy surface for N_2O-He and vibrational band origin shifts for the N_2O-He_N clusters with N=1-40. *J. Chem. Phys.* **137**, 104311 (2012).
2. Bernstein, R. B., editor. *Atom-molecule theory: A Guide for the Experimentalist* (Plenum Press, New York, 1979).
3. Goldflarn, S., Green, S., & Kouri, D. J. Infinite order sudden approximation for rotational energy transfer in gaseous mixtures. *J. Chem. Phys.* **67**, 4149-4161 (1977).
4. Green, S. Computational test of the infinite sudden approximation for excitation of linear rigid rotors by collisions with atoms. *Chem. Phys.* **31**, 425-431 (1978).
5. Liu, W.-K., & Marcus, R. A. On the theory of the relaxation matrix and its application to microwave transient phenomena. *J. Chem. Phys.* **63**, 272-297 (1975).
6. Schwendeman, R. H., & Amano, T. On the comparison of T_1 and T_2 as determined from zero-field and non-zero-field micowave measurements. *J. Chem. Phys.* **70**, 962-973 (1979).
7. Hartmann, J.-M., Boulet, C., & Robert, D. *Collisional Effects on Molecular Spectra. Laboratory Experiments and Models, Consequences for Applications.* (Elsevier, Amsterdam 2008).
8. Brunner, T.A., & Pritchard, D. Fitting laws for rotationally inelastic collisions. In *Dynamics of the Excited State*, K.P. Lawley Ed. (Wiley, New-York, 1982).
9. Green S. On the amount of information in rotational relaxation experiments with application to microwave transient T_1 and T_2 rates. *J. Chem. Phys.* **69**, 4076-4082 (1978).
10. Hartmann, J.-M., & Boulet, C. Quantum and classical approaches for rotational relaxation and nonresonant laser alignment of linear molecules: A comparison for CO_2 gas in the nonadiabatic regime. *J. Chem. Phys.* **136**, 184302 (2012).
11. Bouanich, J.-P., Hartmann, J.-M., Blanquet, G., Walrand, J., Bermejo, D. & Domenech, J.-L. Line-mixing effects in the He- and N_2-broadened Σ–Π infrared Q branches of N_2O. *J. Chem. Phys.* **109**, 6684-6690 (1998).

12. Goldflam, R., Kouri, D.J., & Green S. On the factorization and fitting of molecular scattering information. *J. Chem. Phys.* **67**, 5661-5675 (1977).
13. Weber, M., Sirota, J. M., & Reuter, D.C. l-Resonance Intensity Effects and Pressure Broadening of N_2O at 17 μm. *J. Mol. Spectrosc.* **177**, 211-220 (1996).
14. Ramakrishna, S., & Seideman, T. Intense Laser Alignment in Dissipative Media as a Route to Solvent Dynamics. *Phys. Rev. Lett.* **95**, 113001 (2005).
15. Ramakrishna, S., & Seideman, T. Dissipative dynamics of laser induced nonadiabatic molecular alignment. *J. Chem. Phys.* **124**, 034101 (2006).
